# The chalcone butein from *Rhus verniciflua *Stokes inhibits clonogenic growth of human breast cancer cells co-cultured with fibroblasts

**DOI:** 10.1186/1472-6882-5-5

**Published:** 2005-03-09

**Authors:** Michael Samoszuk, Jenny Tan, Guillaume Chorn

**Affiliations:** 1 Pathology Department, University of California, Irvine Medical Center, Building 10 Route 40, 101 The City Drive, Orange, CA 92868 USA; 2 Biology Department, Stanford University, Stanford, CA USA

## Abstract

**Background:**

Butein (3,4,2',4'-tetrahydroxychalone), a plant polyphenol, is a major biologically active component of the stems of *Rhus verniciflua *Stokes. It has long been used as a food additive in Korea and as an herbal medicine throughout Asia. Recently, butein has been shown to suppress the functions of fibroblasts. Because fibroblasts are believed to play an important role in promoting the growth of breast cancer cells, we investigated the ability of butein to inhibit the clonogenic growth of small numbers of breast cancer cells co-cultured with fibroblasts in vitro.

**Methods:**

We first measured the clonogenic growth of small numbers of the UACC-812 human breast cancer cell line co-cultured on monolayers of serum-activated, human fibroblasts in the presence of butein (2 μg/mL) or various other modulators of fibroblast function (troglitazone-1 μg/mL; GW9662-1 μM; meloxican-1 μM; and 3,4 dehydroproline-10 μg/mL). In a subsequent experiment, we measured the dose-response effect on the clonogenic growth of UACC-812 breast cancer cells by pre-incubating the fibroblasts with varying concentrations of butein (10 μg/ml-1.25 μg/mL). Finally, we measured the clonogenic growth of primary breast cancer cells obtained from 5 clinical specimens with normal fibroblasts and with fibroblasts that had been pre-treated with a fixed dose of butein (2.5 μg/mL).

**Results:**

Of the five modulators of fibroblast function that we tested, butein was by far the most potent inhibitor of clonogenic growth of UACC-812 breast cancer cells co-cultured with fibroblasts. Pre-treatment of fibroblasts with concentrations of butein as low as 2.5 μg/mL nearly abolished subsequent clonogenic growth of UACC-812 breast cancer cells co-cultured with the fibroblasts. A similar dose of butein had no effect on the clonogenic growth of breast cancer cells cultured in the absence of fibroblasts. Significantly, clonogenic growth of the primary breast cancer cells was also significantly reduced or abolished when the tumor cells were co-cultured with fibroblasts that had been pre-treated with a fixed dose of butein.

**Conclusion:**

We conclude that fibroblasts pre-treated with non-toxic doses of butein (a natural herbal compound) no longer support the clonogenic growth of small numbers of primary breast cancer cells seeded into co-cultures. These results suggest that interference with the interaction between fibroblasts and breast cancer cells by the natural herbal compound, butein, should be further investigated as a novel experimental approach for possibly suppressing the growth of micrometastases of breast cancer.

## Background

**Figure 1:**
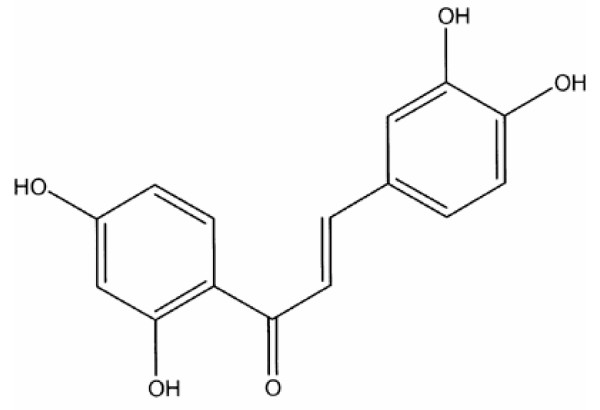
Chemical structure of butein.

Butein (3,4,2',4'-tetrahydroxychalone-Figure 1), a plant polyphenol, is one of the major biologically active components of the bark and stems of *Rhus verniciflua *Stokes. In Far Eastern countries such as Korea, Japan, and China, the compound has been traditionally used for treatment of pain, thrombotic disease, gastritis, stomach cancer, and parasitic infections [1,2]. In Korea, it has also long been used as a food additive [2].

Recently, butein has been shown to possess potent activity against fibroblast function [3], possibly related to its ability to suppress differentiation of fibroblasts to myofibroblasts that are characteristically involved in wound healing [4]. Because fibroblasts and myofibroblasts are now believed to play a critical role in promoting the growth of cancer cells [5,6], we performed this study to determine if butein could suppress the growth of human breast cancer cells co-cultured with fibroblasts by interfering with the function of the fibroblasts.

## Methods

### Clonogenic assay

The UACC-812 human breast cancer cell line (ATCC, Manassas, VA) was passaged in Leibovitz's medium supplemented with 15% fetal calf serum. Normal fibroblasts (CCD-1068SK, ATCC) obtained from the breast of a 65 year old female were passaged at 37°C in minimal essential medium (Eagle's) supplemented with 2 mM L-glutamine, Earle's balanced salt solution (1.5 grams/Liter), sodium bicarbonate, 0.1 mM non-essential amino acids, 1 mL sodium pyruvate, and 10% fetal calf serum in a 5% CO_2 _atmosphere. All cell culture reagents were obtained from ATCC. Our co-culture experiments used confluent monolayers of fibroblasts that had been passaged no more than 21 days. This precaution assured that the fibroblasts were not senescent or transformed.

We seeded 100 UACC-812 breast cancer cells into individual wells of a 96-well cell culture plate containing a confluent monolayer of fibroblasts growing in minimal essential growth medium supplemented as described above. At intervals of 3–4 days, fresh medium was added. After 14 days, the cells were fixed with 70% ethanol for 10 minutes prior to staining for 3 minutes with 0.1% toluidine blue. The wells were then washed with distilled water, and the numbers of colonies of tumor cells containing eight or more confluent cells were counted using inverted microscopy. Each experiment was performed in triplicate, and the means and standard deviations for each treatment and control group were then compared using a two-tailed, unpaired t-test.

### Co-culture with fibroblast modulators (Experiment 1)

A monolayer of normal fibroblasts was seeded with 100 UACC-812 tumor cells/well containing complete culture medium (control) or medium supplemented with various modulators of fibroblast function. The fibroblast modulators that we tested included troglitazone (an activator of PPAR-γ in fibroblasts [7]; 1 μg/mL); GW9662 (an inhibitor of PPAR-γ[8,9]; 1 μM); butein (an inhibitor of myofibroblast differentiation; 2 μg/mL); meloxican (a COX-2 inhibitor in fibroblasts [10,11]; 1 μM); or 3,4 dehydroyproline (an inhibitor of collagen synthesis by fibroblasts [12]; 10 μg/mL). Because butein is relatively insoluble in aqueous solution, it was first dissolved in dimethylsulfoxide to produce a stock solution (10 mg/mL) that was then serially diluted into growth medium to produce the final desired concentrations of butein. At intervals of 3–4 days, old medium was removed and replaced with fresh medium containing the drugs. After 14 days, the numbers of colonies of 12 or more tumor cells were counted as described above. As a control to determine the effects of butein on the clonogenic growth of breast cancer cells in the absence of fibroblasts, we seeded 100 tumor cells into wells without fibroblasts but containing butein (2 μg/mL)

### Co-culture with fibroblasts pre-treated with butein (Experiment 2)

In the next experiment, we sought to determine if pre-treatment of fibroblasts alone with various doses of butein would also inhibit clonogenic growth of breast cancer cells. A monolayer of fibroblasts in individual wells of 96-well plates was incubated for 3 days with growth medium containing serial, 2-fold dilutions of butein ranging from 10 μg/mL to 1.25 μg/mL. The adherent fibroblasts were then washed three times to remove any residual butein, and 100 UACC-812 cells were seeded per well. The co-culture was then incubated in culture medium without butein for 14 days, after which the numbers of colonies of tumor cells were counted as described above.

### Co-culture of primary breast cancer cells with fibroblasts (Experiment 3)

This experiment was performed in order to determine if butein could also suppress the clonogenic growth of primary breast cancer cells obtained directly from clinical specimens of human breast cancer. After obtaining appropriate approval from the Institutional Review Board to perform the study in compliance with the Helsinki Declaration, we aseptically dissected small fragments of tumor tissue from five cases of invasive ductal adenocarcinoma of the breast. These specimens were submitted for routine diagnostic evaluation to the Surgical Pathology Department at UCI Medical Center (Orange, CA). The tissues were carefully minced into small pieces and then digested overnight at 37°C in collagenase II (900 U/mL; Sigma-Aldrich, St. Louis, MO) solution in cell culture medium. Epithelial cells and organoids were then isolated by differential centrifugation of the digest [13], washed, and counted. We then seeded 100 tumor cells onto monolayers of fibroblasts that had been pre-treated with butein (2.5 μg/mL) for 3 days as described in the preceding experiment. The co-cultures were then incubated in culture medium without butein for 14 days, after which the numbers of colonies of tumor cells were counted as described above.

## Results

### Clonogenic assay

**Figure 2:**
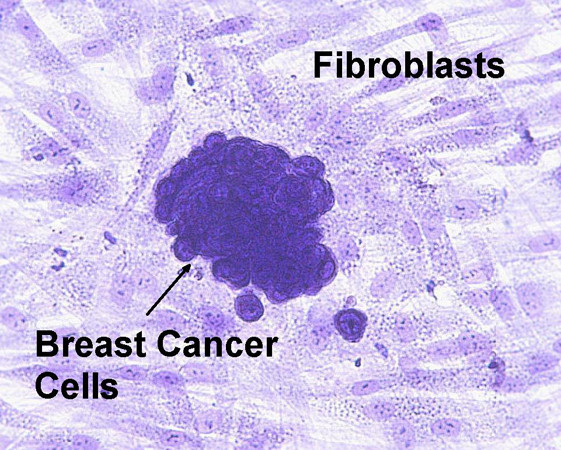
**Representative colony of tumor cells in clonogenic assay. **Breast cancer cells co-cultured with fibroblasts typically formed clusters of confluent cells and were easily distinguished from the fibroblasts when stained with 0.1% toluidine blue. Original magnification 400×.

Co-culture of 100 UACC-812 human breast cancer cells on a monolayer of human fibroblasts without butein treatment typically yielded 50–75 distinct colonies of easily recognizable tumor cells on a background of fibroblasts (Figure 2). If a monolayer of fibroblasts was not used, an average of five small colonies of tumor cells was generally observed.

### Co-culture with fibroblast modulators

**Figure 3:**
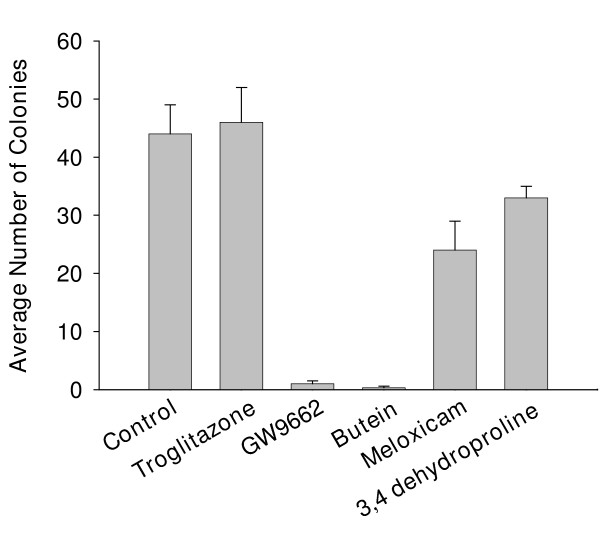
**Co-culture of breast cancer cells and fibroblasts with drugs that modulate fibroblasts. **Compared to the Control (no drugs), butein and GW9662 almost completely eliminated clonogenic growth of the breast cancer cells.

The results of Experiment #1 are presented in Figure 3. Only GW9662 and butein significantly (p < 0.01) reduced clonogenic growth compared to the control (cell culture medium alone with no drug). There was only one small colony of tumor cells visible in one of three wells containing butein. Surviving individual tumor cells were not visible in any of the wells containing butein. Notably, in the absence of fibroblasts, there was an average of 4 colonies of tumor cells regardless of the presence or absence of butein. Thus, butein had no detectable effect on the clonogenic growth of breast cancer cells grown in the absence of fibroblasts.

### Co-culture with fibroblasts pre-treated with butein

**Figure 4:**
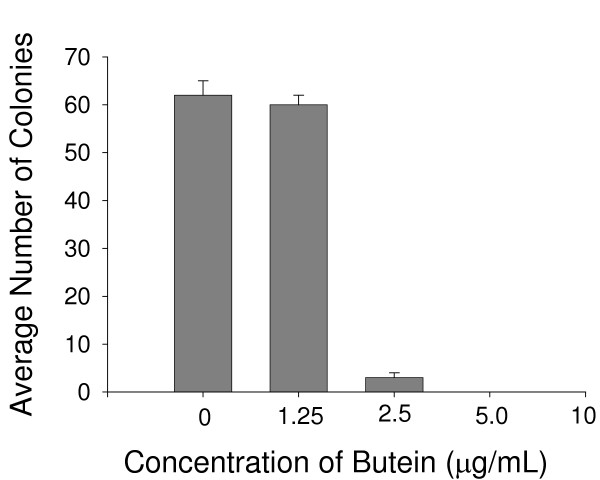
**Co-culture of breast cancer cells with fibroblasts pre-treated with butein. **Pre-treatment of the fibroblasts with butein at concentrations greater than or equal to 2.5 μg/mL completely eliminated or substantially reduced clonogenic growth of co-cultured breast cancer cells, suggesting an indirect mechanism of action that interferes with the interaction between fibroblasts and breast cancer cells.

The results of Experiment #2 are presented in Figure 4. There was no clonogenic growth when 100 UACC-812 breast cancer cells were seeded onto monolayers of fibroblasts that had been pre-treated for 3 days with butein at 10 or 5 μg/mL, and clonogenic growth was significantly reduced at butein concentrations as low as 2.5 μg/mL.

### Co-culture of primary breast cancer cells with fibroblasts pre-treated with butein

The results of Experiment #3 using primary breast cancer cells are presented in Table 1. For each of the 5 clinical specimens that we tested, there were significantly fewer colonies of breast cancer cells when the co-culture was performed in the presence of fibroblasts that had been pre-treated with butein.

**Table 1:** Clonogenic growth of primary breast cancer cells co-cultured with fibroblasts pre-treated with butein (2.5 μg/mL).

Clinical Sample #	Mean number of colonies with butein pre-treatment (1 s.d.)	Mean number of colonies without butein pre-treatment (1 s.d.)
1	0 (0)	13 (4)*
2	0 (0)	36 (9)*
3	0 (0)	6 (4) *
4	3 (2)	12 (5)*
5	7 (4)	29 (8)*

*p value < 0.02 by two-tailed, unpaired t-test comparing means and s.d. of butein pre-treatment versus no pre-treatment

## Discussion

Clonogenic growth of small numbers of breast cancer cells *in vivo *[5,6] as well as in our *in vitro *culture system appears to be critically dependent on the presence of fibroblasts. In this study, we have demonstrated that butein can suppress the clonogenic growth of breast cancer cells *in vitro *through an indirect mechanism that involves interfering with the function of co-cultured fibroblasts. Significantly, the concentration of butein that we found to be effective in these experiments (2.5 μg/ml) does not significantly reduce fibroblast viability [4] and is also non-toxic in animal experiments [1,2]. Moreover, this concentration of butein had no significant effect on the clonogenic growth of breast cancer cells cultured in the absence of fibroblasts.

Previous studies have shown that higher concentrations of butein extract from *Rhus verniciflua *Stokes are directly cytotoxic *in vitro *to human colon adenocarcinoma cells and lymphoma cells [2,14]. Butein has also been shown to inhibit cell growth and induce apoptosis in murine B16 melanoma cells [15]. The mechanism of induction of apoptosis in tumor cells appears to be related to increased caspase-3 activity, decreased Bcl-2 expression, and increased Bax expression [16]. Butein has also been shown to have multiple other activities, including inhibition of epidermal growth factor receptors [17], tyrosine kinase inhibition [18], suppression of E-selectin expression [19], inhibition of tyrosinase enzymes [20], and inhibition of cyclooxygenase-2 [21].

Our results suggest that butein may also suppress the growth of tumor cells through a second, indirect mechanism that does not involve direct toxicity to the tumor cells themselves. At this time, however, the precise molecular mechanisms by which butein interferes with the interaction between fibroblasts and breast cancer cells remain undefined.

## Conclusion

Clonogenic growth of small numbers of breast cancer cells co-cultured with fibroblasts pre-treated with butein is markedly reduced. These results suggest that butein can inhibit the growth of tumor cells through an indirect mechanism that interferes with the interaction between fibroblasts and breast cancer cells. Our results also suggest that the herbal compound butein should be further investigated as a potentially useful experimental approach for suppressing the growth of small numbers of breast cancer cells in early micrometastases.

## List of abbreviations

PPAR-peroxisome proliferation activator receptor

COX-cyclooxygenase

## Competing interests

The authors declare that they have no competing interests.

## Authors' contributions

MKS conceived and designed the study, performed the experiments with the primary tumor cells, analyzed the data from all experiments, and wrote this report. JT and GC performed the other experiments and reported the data.

## Pre-publication history

The pre-publication history for this paper can be accessed here:


